# Supplementary material for: Investigating the procurement system for understanding seasonal influenza vaccine brand availability in Europe
Source: PLoS One. 2021 Apr 8;16(4):e0248943. doi: 10.1371/journal.pone.0248943 (PMC8031425; doi:10.1371/journal.pone.0248943)
Supplement: S1 Appendix — (DOCX) [file pone.0248943.s001.docx]

*Overall aim: to understand vaccine selection, procurement and distribution system, to know which vaccine brand will be used where, in order to set up prospective brand-specific influenza vaccine effectiveness studies.*

**Topic 1. Organization of the national influenza vaccination program (administration)***Aim: to understand if we should focus on national vaccine program or commercial market and to know what type of clinics to go to for prospective studies.*

Background: we would like to understand

1. What proportion of the total country influenza vaccine volume is provided via the national influenza program vs. commercial market?
   1. National influenza vaccination program _______%
   2. Commercial market _______%
2. Which of the following **main** (>20% of total volume) providers administer(s) of the influenza vaccination under the national vaccination program? Roughly, what volume of the national vaccination program is provided by each?
   1. General Practitioners Yes / No _______%
   2. Pharmacist Yes / No _______%
   3. Hospital (inpatients) Yes / No _______%
   4. Hospital (outpatients) Yes / No _______%
   5. Vaccination clinics (permanent) Yes / No _______%
   6. Vaccination clinics (temporarily set up specifically for the influenza vaccination programs) Yes / No _______%
   7. Public health institutes Yes / No _______%
3. Who pays for the vaccines administered under the national vaccination program?
   1. Vaccine recipient
   2. Government
   3. Health insurer
   4. Other, please specify _____________
4. Can risk groups choose health care provider for receipt of vaccine? If yes, does their choice have any impact on reimbursement?
   1. No
   2. Yes
      1. Please specify choice they have: _____________
      2. Please specify impact on reimbursement, if any: _____________
5. Any other comments on the administration of influenza vaccine under the national vaccination program:

_________________________________

**Topic 2. Organization of the procurement of influenza vaccines***Aim:* *To understand how and by whom vaccine brands are chosen and at what level this takes place.*

What type of procurement is in place?

- 1. Public tender
     1. Centrally at national level
     2. Centrally at regional level, nr of regions ______
  2. Preferred provider selection by governmental organization
  3. Direct purchase from manufacturers by health care providers
  4. Indirect from manufacturer through wholesales or distributors
  5. Other, please specify ________________________________

1. What are the main organizations involved in the procurement process of influenza vaccine for the national influenza vaccination program?
   1. Ministry of health
   2. Public health institute
   3. Health insurers
   4. Specific vaccine procurement organizations: ________________________________
   5. Individual healthcare providers: ________________________________
   6. Pooled individual healthcare providers: ________________________________
   7. Wholesalers
   8. Other _____________
2. What is the frequency of vaccine procurement?
   1. Annual
   2. Multiyear contracts: _________________
   3. Other, please specify _________________
3. How many tenders are issued for the country on an annual basis? (e.g. X for X regions) ________________________________
4. Who are the main responsible organization(s) involved in the procurement process for the following activities:
   1. Forecasting of required volume: responsible party: ___________________________
   2. Issuing and managing of the public tenders, responsible party: _______________
   3. Decision making on the selection of vaccines at national level, responsible party: ______
   4. Decision making on the selection of vaccines at regional level, responsible party: ______
   5. Decision making on the selection of vaccines at clinical level, responsible party: ______
   6. Other, please specify _____________
5. Discuss other specifics of procurement system:

_________________________________

1. If there is any significant (>20%) influenza vaccine procurement outside the national influenza vaccine program, how is this organized and for whom (e.g. private health care, nursing home, school programs, hospital etc.)

_________________________________

1. Are there separate procurement systems for different risk populations? (e.g. in categories for age groups)
   1. No
   2. Yes, please specify:

_________________________________

1. Any other comments on the organization or procurement of influenza vaccine under the national vaccination program:

_________________________________

**Topic 3 Timing and stages of the influenza vaccine procurement***Aim: To understand when it is known what vaccine brands will be used (from national down to clinic level)*

1. When (what month) are influenza vaccine volume forecast done (if applicable)?
2. Please describe the timing of the main steps in the influenza vaccine procurement process and timing therefore.
   1. Trigger that starts procurement process: _______
      Timing: _______
   2. First awarded tender/pre-bookings: _______
      Timing: _______
   3. Final awarded tender/final booking: _______
      Timing: _______
3. When do health care providers who administer and/or pharmacies who fill the prescription become aware which vaccine brand(s) will be available for use at their clinic (or pharmacy).

________________________________

1. When are vaccines distributed to the clinical sites or pharmacies (especially in relation to start of vaccination)?

________________________________

1. What is the start of the seasonal vaccination? (including regional variation) ________________________________
2. Any comments on the timing and stages of influenza vaccine procurement under the national vaccination program:
   _________________________________

**Topic 4. Variability and diversity of volume and type of influenza vaccine***Aim: To understand if historical vaccine data can help to predict which vaccine brands will be used in future years*

1. Is there notable and consistent historical variability in the total volume that is tendered? If so, why?
   1. No
   2. Yes, please specify reason: _________________________________
2. Is there notable and consistent historical variability in the brand availability? If so, why?
   1. No
   2. Yes, please specify reason: _________________________________
3. How many **manufacturers** supplying influenza vaccine to the country are considered each year?
   - Average: _______
   - Pre-specified minimum: _______ / Not applicable
   - Pre-specified maximum: _______ / Not applicable
   - Current:
4. How many influenza vaccine **brands** are available each year at a national level?
   - Average: _______
   - Pre-specified minimum: _______ / Not applicable
   - Pre-specified maximum: _______ / Not applicable
   - Current:
5. How many influenza vaccine **types** are available each year at a national level?
   1. Average: _______
   2. Pre-specified minimum: _______ / Not applicable
   3. Pre-specified maximum: _______ / Not applicable
   4. Current:
6. Does the availability of different vaccine brands and types differ at a regional level differ from the availability of vaccines at the national level? i.e. can specific vaccine availability (vaccine brand, vaccine type) be derived from vaccines procured at regional or national level?
7. Are there separate procurement categories by **type** of vaccine?
   1. No
   2. Yes, for the following types:
      1. Conventional
      2. QIV
      3. Adjuvanted
      4. Cell-based
      5. Nasal
      6. Other_______
8. If yes, please describe procurement mechanism

_________________________________

1. How many different vaccine **brands** are generally procured per vaccine **type**?
   1. Conventional: _______
   2. QIV: _______
   3. Adjuvanted: _______
   4. Cell-based: _______
   5. Nasal: _______
   6. Other: _______
2. Any comments on the variability and diversity of volume and type of influenza vaccine under the national vaccination program:
   _________________________________

**Topic 5. Influenza vaccine distribution (optional)***Aim: To understand if distribution data can be used to know which vaccine brand is used where*

1. What are the steps in the distribution process manufacturer to the clinical location where the vaccine will generally be administered, and what organization is responsible for each step (including intermediate logistical parties)?
   1. Step 1: _______
      Organization: _______
   2. Step 2: _______
      Organization: _______
   3. Step 3: _______
      Organization: _______
   4. Step 4: _______
      Organization: _______
2. What party/parties involved in the above distribution chain hold information on which vaccines are available at the clinical site?

_________________________________

1. Is the vaccine distribution organized by region?
   1. Yes, specify nr of regions….
   2. No
2. What is the average number of different distributors which ultimately supply the vaccine to the relevant clinical location where the vaccine will be administered for the country?
   1. Exact number: _______
   2. <10
   3. 10-50
   4. 50-250
   5. >250

**Topic 6. Sources of information on procured vaccine(s)***Aim: To known who holds data on the vaccines brands used at different levels and if it this data is publicly accessible*

1. Who holds which data on the procured vaccines (brand, volume, batch, date of delivery, date of administration) at each level (national, regional, clinic), and how is this data obtained?
2. Is the outcome of the vaccine procurement made public?
   1. No (skip to question 4)
   2. Yes
3. If yes, what information is publicly available, where and when?
4. If any of the above data is not publicly available, would it still be possible to request access?

_________________________________

1. We are also looking for historical data on vaccines used/available. Is this information available to the public? If yes, are you able to provide it / where can it be accessed?

| **Season** | **Vaccine type** | **Vaccine manufacturer awarded tender** | **Vaccine brand name** | **Nr of doses** | **Web link to this information (if available)** |
| --- | --- | --- | --- | --- | --- |
| 2019-2020 (next season) |  |  |  |  |  |
| 2018-2019 |  |  |  |  |  |
| 2017-2018 |  |  |  |  |  |
| 2016-2017 |  |  |  |  |  |
| 2015-2016 |  |  |  |  |  |
| 2014-2015 |  |  |  |  |  |
| 2013-2014 |  |  |  |  |  |

**Topic 7. Other**

- - - 1. Any information source that describes further details of the vaccine procurement, or ways in which use of brand-specific influenza vaccine can be known at clinic level prior to season start
