# Supplementary material for: Investigating the procurement system for understanding seasonal influenza vaccine brand availability in Europe
Source: PLoS One. 2021 Apr 8;16(4):e0248943. doi: 10.1371/journal.pone.0248943 (PMC8031425; doi:10.1371/journal.pone.0248943)
Supplement: S2 Appendix — (PDF) [file pone.0248943.s002.pdf]

On behalf of myself as the rights' holder, I hereby grant permission to use a map created with MapChart (mapchart.net) for a publication in a PLOS journal under a Creative Commons Attribution License (CCAL) CC BY 4.0 (http://creativecommons.org/licenses/by/4.0/).

Minas Giannekas, owner of the map-making website MapChart (mapchart.net)

26 January 2021
