# Supplementary material for: Investigating the procurement system for understanding seasonal influenza vaccine brand availability in Europe
Source: PLoS One. 2021 Apr 8;16(4):e0248943. doi: 10.1371/journal.pone.0248943 (PMC8031425; doi:10.1371/journal.pone.0248943)
Supplement: Attachment — Submitted filename: Response to reviewers_20210122.docx [file pone.0248943.s003.docx]

**PONE-D-20-17950
Investigating the procurement system for understanding seasonal influenza vaccine brand availability in Europe
PLOS ONE

RESPONSE TO REVIEWERS**

| **Reviewers’ comments** | **Authors’ response** |
| --- | --- |
| **Reviewer 1** |  |
| This is a very important topic but I did not feel the paper addressed the core question of whether European countries are getting access to safe and effective influenza vaccines at competitive prices. I am not aware of DRIVE and how this assesses Vaccine Effectiveness (VE) in Europe. Presumably this can be only done "after the fact" i.e. manufacturers will produce their vaccines based on WHO recommendations of anticipated circulating strains and subsequent vaccines may be variably matched resulting in variable VE. | The current work aims to inform and provide essential data in support of the design, feasibility assessment and implementation of post-marketing studies on effectiveness in the EU, and likewise could also support studies with a safety objective.  DRIVE assesses vaccine effectiveness through observational studies, namely test-negative design studies conducted at multiple GP and hospital sites across Europe, and a population-based cohort based on linking national registers (currently in one country).  Results from the 2018/19 season have been published here: <https://www.sciencedirect.com/science/article/pii/S0264410X20310057?via%3Dihub> |
| As a reader, I am more interested to know how many influenza vaccines are potentially available globally, how many are assessed by EMA, how many are licensed by EMA, and what are the differential prices. | Our current research was specifically conducted in the context of establishing a network for vaccine effectiveness monitoring in the EU to respond to a regulatory requirement from the EMA to generate annual brand specific influenza vaccine effectiveness data. For this reason we have limited the assessment to procurement systems in Europe to understand how this determines the availability and diversity of the influenza vaccines licensed by EMA and marketed in Europe.  Pricing was not in scope of the current work as also explained in response to the next question. |
| Then I would like to know which of the 4 procurement mechanisms identified can obtain vaccines at the most competitive prices. Obviously we know that industry often likes to keep their pricing opaque for commercial reasons and we know that likely different countries in Europe many be paying different prices for the same vaccines. This paper highlights 4 main types of procurement mechanisms - from national tender systems choosing only one Product (presumably what is considered the cheapest safe and effective vaccine offered during the tender process). Whereas countries where GPs and Pharmacies buy vaccines from manufacturers have more brands (presumably all safe and effective but likely all at higher prices than the single product purchased through national tender).  The fact that there is no mention of pricing and which of these systems procure vaccines at lower pricing is of concern, since this should be the core interest of health policy makers and regulators i.e. as a policy maker I would be interested to know whether National Tender systems or regional tender systems should be the norm for Europe if it can be shown that those countries using using these systems can purchase lower priced vaccines. Should Europe develop a pooled procurement system similar to the PAHO Revolving Fund? This could be an interesting addition to the discussion. | The ultimate goal of our study was to determine if information on vaccine brands from sources related to vaccine procurement would allow targeted site selection for brand-specific VE studies in DRIVE, to ensure a good coverage of all brands in order to fulfil EMA requirements. Therefore, pricing was outside of the scope of the study.  However, in the discussion we have refer to a study that addresses several of the concerns raised by the reviewers (Wilsdon et al. 2020 Expert Review of Vaccines). This study compares vaccine procurements systems in 4 European countries in terms of price vs. value. They conclude that tenders do indeed lead to lower prices, however they also describe several downsides to tender-based procurement (e.g. in terms of supply sustainability and incentives for innovation). |
| To improve this paper, I think there needs more explanation of the EMA system of licensing, | A sentence on EMA’s different authorization procedures and what this means for vaccine availability has been added to the introduction. |
| more details of the who the 10 national experts are and who the 5 companies are that participated in the interviews. | The experts including name and organization are listed in the acknowledgements. For clarity, the organizations have now been added to the methods section. |
| Given that one of the authors works for a vaccine manufacturer, and that 5 companies contributed data, and that the conclusion seems to be national tender systems have access to less brands, I am concerned that paper gives the impression that "access to more brands is better". | We respectfully disagree with the reviewer, as we wrote the following in the discussion: “Procurement systems which are characterized as national level procurement with multiyear tenders and limited brand diversity, such as in Croatia, the Netherlands, Denmark, Finland and Norway, best allow to prospectively determine vaccine availability.” |
| **Reviewer 2** |  |
| This manuscript is relatively average on describing vaccines procurement across European countries. My suggestion is rather than using telephone interview, it is better to widen and support the writing with each countries' profiles on vaccines procurement. | The reason to conduct expert interviews was that very limited written information was found in the literature and through online searches. This gap in the published literature was also the rationale for conducting the current work. The information on vaccines procured (number of vaccine brands by type) obtained from the expert interviews was substantiated with references, such as the original published tender outcomes (Table 1). |
| It is suggested checklist/tool/assessment used are better to be spell out in this paper. | The interview checklist that was used has been added as Supplementary Material. |
| Extensive descriptions on vaccines procurement can be explained, for example the flow chart on procurement process, stakeholders involve & country's procurement policy.  After all this manuscript is moderately written, can be enhanced by elaborating on aspects mentioned. | We acknowledge that the current work is not exhaustive on all aspects of the procurement policy. Expanding the scope of the work would have however limited our ability to address in sufficient detail the aspects of interest to inform the design and implementation of post-marketing monitoring of vaccine benefit and risk under real-world conditions and fills an important gap in the literature. We have acknowledged this limitation in the discussion and have included a statement to solicit further research. |
